# Mortality burden of pre‐treatment weight loss in patients with non‐small‐cell lung cancer: A systematic literature review and meta‐analysis

**DOI:** 10.1002/jcsm.13477

**Published:** 2024-04-22

**Authors:** Philip D. Bonomi, Jeffrey Crawford, Richard F. Dunne, Eric J. Roeland, Karen E. Smoyer, Mohd Kashif Siddiqui, Thomas D. McRae, Michelle I. Rossulek, James H. Revkin, Lisa C. Tarasenko

**Affiliations:** ^1^ Department of Internal Medicine, Division of Hematology, Oncology and Cell Therapy Rush University Medical Center Chicago IL USA; ^2^ Duke Cancer Institute Duke University Medical Center Durham NC USA; ^3^ Department of Medicine and Wilmot Cancer Institute, Division of Hematology/Oncology University of Rochester Medical Center Rochester NY USA; ^4^ Knight Cancer Institute Oregon Health and Science University Portland OR USA; ^5^ Curo Envision Pharma Group Philadelphia PA USA; ^6^ EBM Health Consultants New Delhi Delhi India; ^7^ Internal Medicine Business Unit, Global Product Development Pfizer Inc New York NY USA; ^8^ Internal Medicine Research Unit, Worldwide Research, Development and Medical Pfizer Inc Cambridge MA USA; ^9^ Internal Medicine Research Unit, Clinical Development Pfizer Inc Cambridge MA USA; ^10^ Global Medical Affairs Pfizer Inc New York NY USA

**Keywords:** cachexia, meta‐analysis, muscle wasting, non‐small‐cell lung cancer, systematic literature review, weight loss

## Abstract

Cachexia, with weight loss (WL) as a major component, is highly prevalent in patients with cancer and indicates a poor prognosis. The primary objective of this study was to conduct a meta‐analysis to estimate the risk of mortality associated with cachexia (using established WL criteria prior to treatment initiation) in patients with non‐small‐cell lung cancer (NSCLC) in studies identified through a systematic literature review. The review was conducted according to PRISMA guidelines. Embase® and PubMed were searched to identify articles on survival outcomes in adult patients with NSCLC (any stage) and cachexia published in English between 1 January 2016 and 10 October 2021. Two independent reviewers screened titles, abstracts and full texts of identified records against predefined inclusion/exclusion criteria. Following a feasibility assessment, a meta‐analysis evaluating the impact of cachexia, defined per the international consensus criteria (ICC), or of pre‐treatment WL ≥ 5% without a specified time interval, on overall survival in patients with NSCLC was conducted using a random‐effects model that included the identified studies as the base case. The impact of heterogeneity was evaluated through sensitivity and subgroup analyses. The standard measures of statistical heterogeneity were calculated. Of the 40 NSCLC publications identified in the review, 20 studies that used the ICC for cachexia or reported WL ≥ 5% and that performed multivariate analyses with hazard ratios (HRs) or Kaplan–Meier curves were included in the feasibility assessment. Of these, 16 studies (80%; *n* = 6225 patients; published 2016–2021) met the criteria for inclusion in the meta‐analysis: 11 studies (69%) used the ICC and 5 studies (31%) used WL ≥ 5%. Combined criteria (ICC plus WL ≥ 5%) were associated with an 82% higher mortality risk versus no cachexia or WL < 5% (pooled HR [95% confidence interval, CI]: 1.82 [1.47, 2.25]). Although statistical heterogeneity was high (I^2^ = 88%), individual study HRs were directionally aligned with the pooled estimate, and there was considerable overlap in CIs across included studies. A subgroup analysis of studies using the ICC (HR [95% CI]: 2.26 [1.80, 2.83]) or WL ≥ 5% (HR [95% CI]: 1.28 [1.12, 1.46]) showed consistent findings. Assessments of methodological, clinical and statistical heterogeneity indicated that the meta‐analysis was robust. Overall, this analysis found that ICC‐defined cachexia or WL ≥ 5% was associated with inferior survival in patients with NSCLC. Routine assessment of both weight and weight changes in the oncology clinic may help identify patients with NSCLC at risk for worse survival, better inform clinical decision‐making and assess eligibility for cachexia clinical trials.

## Introduction

Cachexia is a complex metabolic syndrome characterized by a loss of appetite (anorexia), involuntary weight loss and decreased skeletal muscle mass that cannot be fully reversed by conventional nutritional support.^1^, ^2^, ^3^ Often exacerbated by cancer treatment modalities, cachexia is associated with fatigue, functional impairment, decreased treatment intensity, increased treatment toxicity, a poorer quality of life and reduced survival.^3^, ^4^

Although commonly associated with cancer, cachexia prevalence rates vary markedly according to cancer type,^5^, ^6^, ^7^, ^8^ disease stage,^6^, ^8^ patient setting (inpatient vs. outpatient),^9^ patient sex,^10^ comorbidities^11^ or the use of anti‐cancer therapies.^12^ Various definitions and criteria used to identify cancer cachexia confound the reporting of its prevalence rates,^1^, ^13^, ^14^, ^15^ limiting the ability to synthesize prevalence data across different studies. A landmark international consensus definition for cancer cachexia that included diagnostic criteria and a disease classification system^1^ represented an important step in guiding the identification and clinical management of cancer cachexia. These consensus diagnostic criteria for cancer cachexia consisted of involuntary weight loss > 5% of a patient's baseline weight over the previous 6 months; or weight loss > 2% over the previous 6 months and body mass index (BMI) < 20.0 kg/m^2^; or weight loss > 2% over the previous 6 months and loss of skeletal muscle mass consistent with sarcopenia.^1^

Despite the publication of these consensus diagnostic criteria over a decade ago, few studies have investigated the impact of cancer cachexia on patient survival according to these consensus criteria. Instead, research in this space has focused on single features of cachexia or a subset of these factors. Published studies are typically retrospective in nature, in which available archived data often miss one component of the criteria (reliable weight loss data, BMI or muscle mass assessment). Heterogeneity in the body mass and body composition metrics assessed, as well as overall study quality, often hamper the pooling of cancer cachexia studies.^16^ Consequently, meta‐analyses of cancer cachexia data often focus on individual features of cachexia, in particular muscle mass quantity.^17^, ^18^, ^19^, ^20^

Current estimates place lung cancer as the second most commonly diagnosed cancer and the leading cause of cancer deaths globally; an estimated 2.2 million new cases of lung cancer and 1.8 million lung cancer deaths occurred worldwide in 2020.^21^ The reported prevalence of cachexia in the context of lung cancer ranges from around 30% to upwards of 80%.^6^, ^7^, ^8^, ^9^ With non‐small‐cell lung cancer (NSCLC) accounting for ~85% of all lung cancer diagnoses,^22^ the potential for cachexia to adversely affect patient outcomes in NSCLC is significant. Hence, an updated assessment of the relationship between cachexia and survival in NSCLC is warranted and may help identify opportunities for improving patient care. To this end, a systematic literature review (SLR) and meta‐analysis were conducted to evaluate the strength of the evidence base and to calculate a single summary estimate of the impact of cachexia (defined primarily by weight loss ≥ 5%) on survival in patients with NSCLC by integrating the findings from multiple studies.

### Objectives

The primary objective of this SLR and meta‐analysis was to comprehensively evaluate the risk of mortality associated with cachexia or weight loss in patients with NSCLC as identified in published studies. Based on the findings from an initial feasibility assessment, meta‐analyses of studies reporting the impact of cachexia, as defined by the international consensus diagnostic criteria,^1^ or of previous weight loss ≥ 5% (where the timeframe for weight loss was not specified), on overall survival in patients with NSCLC were conducted.

## Methods

### Conduct of the systematic literature review

The SLR was conducted according to predefined protocol and in accordance with the Preferred Reporting Items for Systematic Reviews and Meta‐Analyses (PRISMA) 2020 statement^23^ and the PRISMA Protocol (PRISMA‐P) guidelines.^24^ The SLR protocol was prospectively registered in the International Prospective Register of Systematic Reviews (PROSPERO); study registration was received on 24 January 2022 (registration number: CRD42022284170).

### Data sources and search strategy for the systematic literature review

Literature searches conducted as part of a broader SLR on cachexia and weight loss in selected solid‐tumour cancers were used to identify studies in patients with NSCLC. Searches were run in the Embase and PubMed databases on 11 October 2021. Detailed search strategies are presented in *Tables* *S1* and *S2*. Initial searches encompassed the period 1 January 2011 to 10 October 2021. However, owing to the large number of records identified, the results were narrowed to the 5‐year period prior to the search date (1 January 2016 to 10 October 2021). Moreover, by focusing on these most recent data, the results of the meta‐analysis would be more reflective of current treatment patterns (including immunotherapy and targeted therapies) and survivorship in NSCLC. A manual search of reference lists from retrieved publications and relevant reviews was also conducted.

### Inclusion criteria for the meta‐analysis

The eligible study populations, interventions, comparators, outcomes and study designs (PICOS) for inclusion in the meta‐analysis are presented in *Table* *1*. Studies evaluating overall survival and cachexia or weight loss in adult patients with NSCLC (any stage) published in English‐language, peer‐reviewed journals between 1 January 2016 and 10 October 2021 were eligible for inclusion (*Table* *1*). Studies in broader lung cancer populations where the proportion of patients with NSCLC was not specified were included based on the epidemiology of lung cancer (~85% of patients would have NSCLC^22^).

**Table 1:** Summary of the PICOS criteria for inclusion of studies in the meta‐analysis

Parameter	Included	Excluded
Populations	Studies in adult patients with NSCLC^a^ and cachexia or at risk of cachexia, as defined by the IC diagnostic criteria for cachexia or with weight loss ≥ 5% (see *Table* *2*)	Studies in paediatric patients Studies without results specific to NSCLC Studies in other cancers Studies without patients with cachexia or weight loss Studies in patients unable to orally consume food or who are restricted to liquid nutrition
Interventions	Any or none other than those excluded	Interventions intended for weight loss Parenteral or enteral nutrition Surgery or resection
Comparators	Any or none other than those excluded	Interventions intended for weight loss Parenteral or enteral nutrition Surgery or resection
Outcomes	OS comparing the population with vs. without cachexia or weight loss OS presented based on multivariate analyses OS reported either as HR with the measure of variance (95% CI, SE, SD or *P* value) or as Kaplan–Meier curves	Studies not reporting OS for patients with NSCLC and cachexia or weight loss Studies reporting univariate or unadjusted analyses Studies reporting weight loss as a continuous variable
Study designs	Randomized or non‐randomized clinical trials Retrospective or prospective real‐world/observational studies Study types as above with ≥100 patients in total	Pre‐clinical, animal or case studies, economic modelling studies Notes, commentaries, editorials, opinions or letters Meta‐analyses or reviews^b^ Studies with <100 patients in total
Other limits	Publications in English and published between 1 January 2016 and 10 October 2021	Publications not in English or published prior to 2016

Abbreviations: CI, confidence interval; HR, hazard ratio; IC, international consensus; NSCLC, non‐small‐cell lung cancer; OS, overall survival; PICOS, populations, interventions, comparators, outcomes and study designs; SD, standard deviation; SE, standard error.

^a^ Studies in broad lung cancer populations were included based on the assumption that the patient populations reflected the epidemiology of lung cancer (~85% of patients would have NSCLC^22^).

^b^ Reviews were excluded, but reference lists of relevant systematic reviews were screened for primary sources.

Patient populations of interest were further defined according to one of two sets of criteria for cachexia or weight loss identified at baseline (*Table* *2*). First, the international consensus diagnostic criteria for cachexia^1^ consist of weight loss > 5% over the previous 6 months; or weight loss > 2% over the previous 6 months and BMI < 20.0 kg/m^2^; or weight loss > 2% over the previous 6 months and evidence of muscle depletion (sarcopenia) according to standardized body composition measurements and reference values (see *Table* *2*). Second, patients with previous weight loss ≥ 5% without specifying the timeframe for the weight loss were included in this analysis (*Table* *2*). Only those studies that reported weight loss at baseline or study entry (prior to treatment initiation) were included in the meta‐analysis.

**Table 2:** Populations of interest: Cancer cachexia or weight loss

IC diagnostic criteria for cancer cachexia
Diagnostic criteria as described in Fearon et al.^1^:
Weight loss > 5% in the previous 6 months, OR
Weight loss > 2% in the previous 6 months AND one of the following:
• BMI < 20.0 kg/m^2^, OR
• Evidence of muscle depletion (sarcopenia), such as:
• Appendicular skeletal muscle index determined by dual‐energy X‐ray absorptiometry (men <7.26 kg/m^2^; women <5.45 kg/m^2^)
• Mid upper‐arm muscle area determined by anthropometry (men <32 cm^2^; women <18 cm^2^)
• Lumbar skeletal muscle index determined by CT imaging (men <55 cm^2^/m^2^; women <39 cm^2^/m^2^)
• Whole body fat‐free muscle mass index without bone determined by bioelectrical impedance (men <14.6 kg/m^2^; women <11.4 kg/m^2^)
• Absolute muscularity below the 5th percentile

Broader diagnostic criterion for unintentional weight loss
Weight loss ≥ 5%, but IC criteria otherwise undefined or not met

Abbreviations: BMI, body mass index; CT, computed tomography; IC, international consensus.

### Study selection, data extraction and data reporting

**Figure 1:**
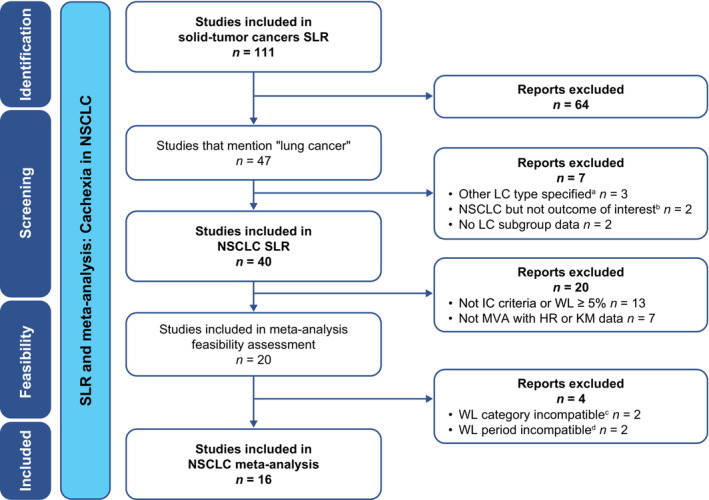
PRISMA diagram of the literature‐screening process for the NSCLC SLR and meta‐analysis. ^a^LC types were SCLC (*n* = 2) or malignant pleural mesothelioma (*n* = 1). ^b^Outcomes were PFS or weight gain. ^c^WL analysed as quartiles or as a continuous variable in MVA. ^d^WL assessment periods were during treatment rather than at baseline. HR, hazard ratio; IC, international consensus; KM, Kaplan–Meier; LC, lung cancer; MVA, multivariate analysis; NSCLC, non‐small‐cell lung cancer; PFS, progression‐free survival; PRISMA, Preferred Reporting Items for Systematic Reviews and Meta‐Analyses; SCLC, small‐cell lung cancer; SLR, systematic literature review; WL, weight loss.

Study selection was performed through consecutive stages that included the removal of duplicate records and record prescreening in EndNote, and title/abstract and full‐text screening against the study inclusion and exclusion criteria. These processes were conducted by two independent reviewers, with screening discrepancies resolved by consensus. For the current SLR and meta‐analysis, a subset of studies conducted in patients with NSCLC was then identified for further review and analysis. The process of identifying publications and reasons for exclusion are presented in *Figure* *S1* (for the broader SLR on cachexia and weight loss in selected solid‐tumour cancers) and *Figure* *1* (for the NSCLC SLR and meta‐analysis). Data from the included publications were extracted by one researcher into prespecified data extraction grids in Microsoft Excel and quality‐checked by a second researcher against the original publications. Data for the two populations with cachexia or weight loss (*Table* *2*) were reported separately. Study and population characteristics, and outcomes data for patients with versus without cachexia or weight loss, were reported where available.

### Meta‐analysis feasibility assessment

A meta‐analysis feasibility assessment was conducted in line with Cochrane recommendations^25^ to evaluate variability in study characteristics (methodological heterogeneity) and patient, disease and outcome characteristics (clinical heterogeneity) across studies. This qualitative assessment determined the suitability of conducting a meta‐analysis and the sensitivity and subgroup analyses required to account for any heterogeneity identified across the included studies. The findings of the feasibility assessment were used to guide the subsequent steps of the meta‐analysis, including the selection of appropriate statistical models.

### Statistical analyses

Data synthesis was performed using pairwise random‐effects meta‐analyses to generate pooled hazard ratios (HRs) and 95% confidence intervals (CIs). A fixed‐effects meta‐analysis was also run alongside the base‐case random‐effects meta‐analysis. Quantitative tests of statistical heterogeneity included the chi‐square test and the I^2^ statistic.^26^, ^27^ A Galbraith plot was generated to assess variability among effect sizes and identify any outlier studies.^28^ In addition to sensitivity and subgroup analyses, the validity of the base‐case meta‐analysis was assessed by cumulative meta‐analyses to identify any time‐varying effects across studies and by a meta‐influence analysis to assess the relative influence of each study on the results. A funnel plot was used to assess any publication bias.^29^ All analyses were conducted using Stata statistical software (Version 17).

For studies that did not provide point estimates, data from Kaplan–Meier curves (survival probability over time, number of events and numbers at risk) were extracted using Engauge Digitizer software (Version 12.1). Pseudo‐individual patient data (IPD) were generated using the Guyot algorithm and accompanying R code.^30^ The pseudo‐IPD for each arm were used to estimate the HRs and associated CIs.

### Study quality assessment

All studies identified for inclusion in the final meta‐analysis were longitudinal observational studies in design. Hence, study quality was assessed and checked by independent researchers using the Newcastle–Ottawa Scale^31^ for risk of bias.

## Results

### Study selection

A total of 7187 records were identified from the database searches covering the period from 1 January 2011 to 10 October 2021 (*Figure* *S1*). Following the removal of duplicates, articles published prior to 2016 and non‐relevant studies, 3282 records passed through title and abstract screening, and 191 articles were retained for full‐text review. Overall, 111 publications were identified as part of the broader SLR on the association between cachexia (defined per international consensus criteria) or weight loss and survival in patients with selected solid‐tumour cancers (*Figure* *S1*). Of these, 40 studies were identified for inclusion in the NSCLC SLR and feasibility assessment (*Figure* *1*).

### Meta‐analysis feasibility assessment

Of the 40 NSCLC studies identified, 20 studies^32^, ^33^, ^34^, ^35^, ^36^, ^37^, ^38^, ^39^, ^40^, ^41^, ^42^, ^43^, ^44^, ^45^, ^46^, ^47^, ^48^, ^49^, ^50^, ^51^ that used the international consensus criteria for cachexia or reported weight loss ≥ 5%, and that performed multivariate analyses with HRs or Kaplan–Meier curves, were included in the meta‐analysis feasibility assessment to identify any methodological and clinical heterogeneity across the studies (*Figure* *1*). Two studies were considered unsuitable for inclusion in the meta‐analysis due to differences in the categorization of weight loss used in the multivariate analysis compared with the other studies, namely, the use of weight loss percentage quartiles^47^ or weight loss percentage as a continuous variable,^46^ rather than as a dichotomous categorical variable (≥ 5% vs. < 5%). A further two studies were excluded due to differences in the assessment periods for weight loss, namely, weight loss during radiotherapy rather than at baseline or study entry.^37^, ^45^

Study and patient characteristics for the remaining 16 studies are shown in *Table* *3*. All studies were observational in design (*n* = 2 prospective; *n* = 14 retrospective), and the definition of overall survival was similar across studies. Heterogeneity in study characteristics was observed for the geographic region, the time period of data collection, follow‐up time and treatment type used. Heterogeneity in patient characteristics was observed for age, gender, performance status and disease stage.

**Table 3:** Design and patient characteristics of non‐small‐cell lung cancer studies identified during the feasibility analysis for inclusion in the meta‐analysis (*n* = 16)

Author; Year; Country	Cancer type; Treatment type	Study design; Sample size; Study period; Follow‐up	Definition of cachexia or weight loss	Age; Male gender	Disease stage; Performance status^a^	Definition of overall survival outcome
Gannavarapu et al.; 2018; NR (US assumed)^32^	Multiple cancers incl. NSCLC; Tx NR	Retrospective observ.; *n* = 3180 (*n* = 1369 with NSCLC); 2006–2013; >5 years	IC criteria: Overt WL defined as UWL > 5% in prior 6 months and BMI ≥ 20 kg/m^2^, or UWL > 2% and BMI < 20 kg/m^2^	Median: 62 years; 57.4% (overall cohort)	I/II: 391 (28.6%) III/IV/R: 958 (70.0%); PS NR (NSCLC cohort)	Time between cancer diagnosis and death with pts censored at time of last clinic visit (if lost to FU) or EOS period
Ganti et al.; 2019; US^33^	NSCLC; Mixed Tx modalities	Retrospective observ.; *n* = 963; 1991–2011; FU NR	WL ≥ 5% but not meeting IC criteria: WL ≥ 5% in prior 3 months or ≥10% in prior 6 months	Category: ≥70 years; 33.4%	I/II: 0 (0%) III/IV/R: 963 (100%); 0–1: 883 (91.7%) ≥2: 80 (8.3%)	Time between random assignment or registration and death resulting from any cause
Holmes et al.; 2017; Canada^34^	NSCLC; Radiotherapy	Retrospective observ.; *n* = 335^b^; 1994–2013; Median: 4.7 years	WL ≥ 5% but not meeting IC criteria: WL < 5%, 5–10% or >10%	Median: 74.7 years; 40%	I/II: 335 (100%) III/IV/R: 0 (0%); 0–1: 251 (74.9%) ≥2: 84 (25.1%)	Time between biopsy (diagnosis) and death of any cause
Jo et al.; 2021^c^; Japan^35^	NSCLC; Immunotherapy	Retrospective observ.; *n* = 133; 2017–2020; >2 years	IC criteria: Involuntary WL > 5% in prior 6 months, or BMI < 20 kg/m^2^ and WL > 2%	Av. median^d^: 64 years; 66.2%	I/II: 0 (0%) III/IV/R: 133 (100%); 0–1: 114 (85.7%) ≥2: 19 (14.3%)	Time between date of Tx initiation and date of death
Jouinot et al.; 2020; France^36^	NSCLC; Tx NR	Prospective observ.; *n* = 144^b^; 2012–2017; Median: 0.82 years	IC criteria: WL in prior 6 months ≤ 5% or >5%	Mean: 64.2 years; 63.2%	I/II: 0 (0%) III/IV/R: 144 (100%); 0–1: 81 (56.3%) ≥2: 63 (43.8%)	Time between evaluation and death or last FU visit
Lee et al.; 2020; US^38^	NSCLC; Immunotherapy	Retrospective observ.; *n* = 106; 2014–2017; 2 years^e^	IC criteria: Significant WL at presentation, defined as WL ≥ 5% in prior 6 months	Mean: 68.6 years; 59.4%	I/II: 0 (0%) III/IV/R: 96 (90.6%); 0–1: 69 (65.1%) ≥2: 35 (33.0%)	Time between initial Tx (time from first dose of immunotherapy) and the event endpoint (death) with pts censored at the EOS period
Miyawaki et al.; 2020; Japan^39^	NSCLC; Immunotherapy	Retrospective observ.; *n* = 108; 2016–2019; Median: 1.51 years	IC criteria: UWL ≥ 5% during 6 months prior to initiation of Tx	Median: 67 years; 75.9%	I/II: 0 (0%) III/IV/R: 108 (100%); 0–1: 108 (100%) ≥2: 0 (0%)	Time between start of Tx and event endpoint (death)
Moore et al.; 2020; Canada^40^	NSCLC; Mixed Tx modalities	Retrospective observ.; *n* = 290^b^ (non‐surgical cohort); 2005–2012; >5 years	WL ≥ 5% but not meeting IC criteria: WL < 5%, 5–10% and >10%	Av. median^d^: 75.5 years; 51.4%	I/II: 290 (100%) III/IV/R: 0 (0%); 0–1: 146 (50.3%) ≥2: 133 (45.9%)	Time between date of diagnosis and death
Morimoto et al.; 2021; Japan^41^	NSCLC; Mixed Tx modalities	Retrospective observ.; *n* = 196; 2019–2019; Median: 1.15 years	IC criteria: WL > 5% within 6 months prior to Tx initiation, or WL > 2% with BMI < 20 kg/m^2^, and abnormal biochemistry^f^; based on the IC criteria^1^ and Evans et al.^14^	Median: 69 years; 72.4%	I/II: 0 (0%) III/IV/R: 196 (100%); 0–1: 190 (96.9%) ≥2: 6 (3.1%)	Time between chemoimmunotherapy initiation and death
Moumtzi et al.; 2016; Greece^42^	NSCLC; Chemotherapy ± radiotherapy	Retrospective observ.; *n* = 1156; 1987–2013; ~2 years^g^	WL ≥ 5% but not meeting IC criteria: WL ≥ 5% in prior 3 months	Median: 62 years; 88.1%	I/II: 0 (0%) III/IV/R: 1156 (100%); PS NR	Time between start of Tx and date of death from any cause or date of last contact with the pt
Patel et al.; 2017; US^43^	NSCLC (metastatic EGFR mutant); Targeted therapy (TKI)	Retrospective observ.; *n* = 189; 2004–2013; Median: 2.1 years	WL ≥ 5% but not meeting IC criteria: WL > 5% vs. WL 0–5%	Median: 60 years; 29.6%	I/II: 31 (16.4%) III/IV/R: 158 (83.6%); <80: 34 (18.0%) ≥80: 155 (82.0%) (Karnofsky PS)	NR
Roch et al.; 2020; France^44^	NSCLC; Immunotherapy	Retrospective observ.; *n* = 142^b^; 2015–2017; Median: 0.5 years	IC criteria: Cachexia defined as a body WL ≥ 5% in prior 6 months	Mean: 63.5 years; 65.5%	I/II: 0 (0%) III/IV/R: 142 (100%); 0–1: 94 (66.2%) ≥2: 48 (33.8%)	Time between first administration of immunotherapy and death due to any cause
Topkan et al.; 2018; Turkey^48^	NSCLC (SCC); Chemoradiotherapy	Retrospective observ.; *n* = 789; 2007–2013; Median: 1.91 years	IC criteria: WL > 5% in prior 6 months	Median: 64.3 years; 66.2%	I/II: 0 (0%) III/IV/R: 789 (100%); 0–1: 789 (100%) ≥2: 0 (0%)	Time interval between first day of C‐CRT and death or last visit
Topkan et al.; 2020; Turkey^49^	NSCLC; Chemoradiotherapy	Retrospective observ.; *n* = 145; 2007–2014; Median: 1.8 years	IC criteria: WL > 5% in 6 months prior to initiation of Tx in pts with BL BMI ≥ 20 kg/m^2^	Median: 38 years; 75.9%	I/II: 0 (0%) III/IV/R: 145 (100%); 0–1: 145 (100%) ≥2: 0 (0%)	Time interval between first day of C‐CRT and date of death/last visit
Wang et al.; 2017; China^50^	NSCLC; Radiotherapy ± chemotherapy	Retrospective observ.; *n* = 140; 2009–2012; Median: 2.17 years	IC criteria: WL ≥ 5% within 6 months after the initial hospital visit	Median: 60 years; 75%	I/II: 42 (30%) III/IV/R: 98 (70%); 0–1: 140 (100%) ≥2: 0 (0%)	Time between histological diagnosis and death from any cause, or the date of the last FU
Watte et al.; 2018; Brazil^51^	NSCLC; Chemotherapy and/or radiotherapy	Prospective observ.; *n* = 104; 2014–2016; 0.25 years	IC criteria: WL in 6 months prior to diagnosis, stratified into WL ≥ 5%, ≥10% and ≥15% categories	Median: 63 years; 60.6%	I/II: 0 (0%) III/IV/R: 104 (100%); Median PS: 2	Time between histological diagnosis and death or until the end of the third month of FU

Abbreviations: Av., average; BL, baseline; BMI, body mass index; C‐CRT, concurrent chemoradiotherapy; CRP, C‐reactive protein; ECOG, Eastern Cooperative Oncology Group; EGFR, epidermal growth factor receptor; EOS, end of study; FU, follow‐up; IC, international consensus; NR, not reported; NSCLC, non‐small‐cell lung cancer; observ., observational; OS, overall survival; PS, performance status; pts, patients; R, recurrent; SCC, squamous cell carcinoma; TKI, tyrosine kinase inhibitor; Tx, treatment; US, United States; UWL, unintentional weight loss; WL, weight loss.

^a^ Performance status was based on the ECOG scale in all studies except for Patel et al.,^43^ which used the Karnofsky scale.

^b^ Sample size with NSCLC at baseline; for some studies, this is higher than the sample size with NSCLC and OS data.

^c^ E‐publication date: June 2021; final publication date: February 2022.

^d^ Average median was the average of reported median age from two cohorts.

^e^ Assumed to be >2 years, based on the follow‐up time reported in Kaplan–Meier curves for OS.

^f^ Abnormal biochemistry: CRP > 0.5 mg/dL, haemoglobin < 12 g/dL or serum albumin < 3.2 g/dL.

^g^ Assumed to be ~2 years, based on the median OS (<2 years).

Based on this feasibility assessment, a meta‐analysis using a random‐effects model, which considers variability across studies, incorporating the 16 studies as the base case was conducted. The impact of heterogeneity was evaluated through the sensitivity and subgroup analyses listed in *Table* *S3*, which included an additional subgroup analysis based on the two sets of criteria for cachexia or weight loss used to define the study populations of interest (the international consensus criteria for cachexia or weight loss ≥ 5% but the international consensus criteria otherwise not met), as well as cumulative analyses of time‐varying effects and study influence. An assessment of the impact of treatment on the mortality associated with cachexia or weight loss ≥ 5% was not conducted. Across the studies, cachexia or weight loss was consistently associated with significantly higher mortality, regardless of the type of treatment administered. Consequently, segregating the studies by treatment type and conducting a subgroup analysis would not alter the conclusion of the base‐case analysis, given that a significant impact was observed across all treatment types.

### Study quality assessment

The results of the risk‐of‐bias assessment using the Newcastle–Ottawa Scale^31^ are shown in *Table* *S4* for the 16 studies identified for inclusion in the meta‐analysis. Fourteen studies (87.5%) scored 8 or 9 points, indicating a low risk of bias, and the remaining two studies (12.5%) scored 7 points, indicating a medium risk of bias. There were no studies with scores indicating a high risk of bias; hence, there was no exclusion of studies from the meta‐analysis on this basis.

### Association between cachexia or weight loss and overall survival

#### Base‐case analysis

**Figure 2:**
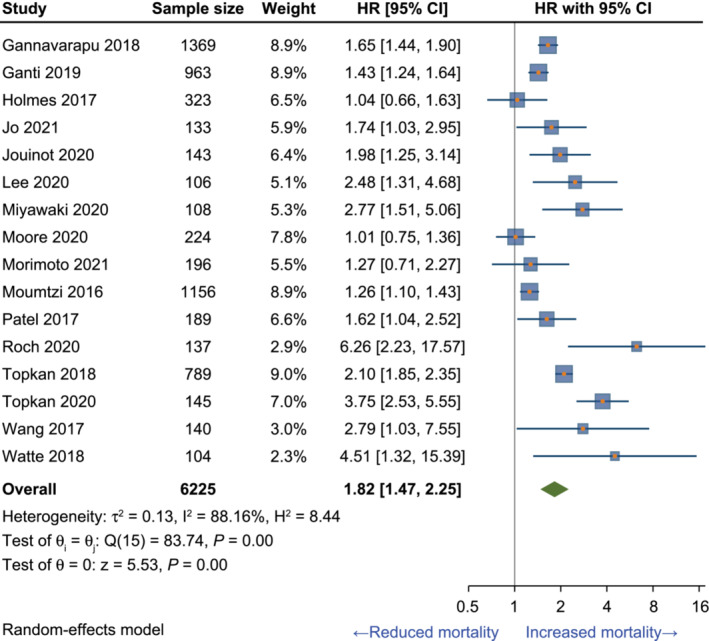
Meta‐analysis of the association between cachexia or weight loss ≥ 5% and overall survival in NSCLC: Base‐case random‐effects model. Sample size reflects patients with NSCLC and OS data, which for some studies is less than the number of patients with baseline data. CI, confidence interval; H^2^, homogeneity statistic; HR, hazard ratio; I^2^, heterogeneity statistic; NSCLC, non‐small‐cell lung cancer; OS, overall survival; Q, Cochrane Q (chi‐square statistic); T^2^, tau‐square; z, normality distribution; θ, overall effect estimate.

**Figure 3:**
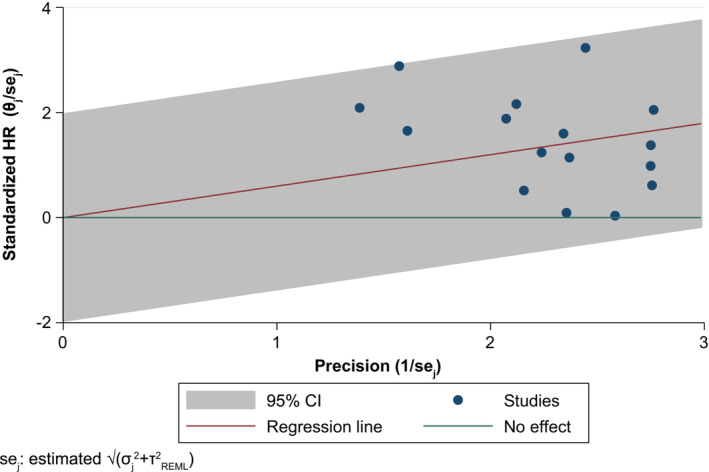
Galbraith plot analysis for the base‐case meta‐analysis of the association between cachexia or weight loss ≥ 5% and overall survival in non‐small‐cell lung cancer. θ and σ represent the study‐specific effect size and its standard error. Navy circles represent study‐specific log HR divided by sigma (θ/σ) against study precisions 1/σ. CI, confidence interval; HR, hazard ratio; REML, restricted maximum likelihood; se, standard error; θ, overall effect estimate.

The 16 studies considered suitable for inclusion in the base‐case meta‐analysis comprised 6309 patients with NSCLC (*Table* *3*), of whom 6225 had overall survival data. The results of the base‐case meta‐analysis using a random‐effects model found that, across the 16 studies, patients with cachexia or weight loss ≥ 5% had a significantly higher risk of mortality versus those with no cachexia or weight loss < 5% (pooled HR [95% CI]: 1.82 [1.47, 2.25]) (*Figure* *2*). Statistical heterogeneity among studies was high (I^2^ = 88.2%). However, as the HRs of the individual studies and the pooled estimate were directionally aligned, and there was considerable overlap in the CIs across individual studies, the meta‐analysis results were considered robust. Additionally, an assessment of heterogeneity across studies using a Galbraith plot did not identify any substantial variability among effect sizes (*Figure* *3*). Consistent results were found with an analysis using a fixed‐effects model, where cachexia or weight loss ≥ 5% was also associated with a higher risk of mortality than no cachexia or weight loss < 5% (pooled HR [95% CI]: 1.63 [1.53, 1.73]).

#### Sensitivity analyses

The validity of the base‐case meta‐analysis was assessed by accounting for differences in population characteristics using sensitivity analyses after excluding outlier studies on the basis of age, gender, performance status and disease stage (*Table* *S3*). A statistically significant association between cachexia or weight loss ≥ 5% and inferior survival, compared with no cachexia or weight loss < 5%, was observed for all variables assessed (*Table* *4* and *Figure* *S2*), indicating that the results of the base‐case meta‐analysis were robust and generalizable despite variability in these characteristics between studies.

**Table 4:** Sensitivity and subgroup meta‐analyses of the association between cachexia or weight loss ≥ 5% and overall survival in non‐small‐cell lung cancer: Random‐effects model

Type of analysis/variable	No. of studies	Sample size	HR (95% CI)	I^2^ (%)
Base‐case analysis (REM)	16	6225	1.82 (1.47, 2.25)	88.2
Sensitivity analyses				
Age	15	6080	1.68 (1.40, 2.01)	81.5
Gender	13	3917	2.00 (1.53, 2.61)	85.7
Performance status	12	5615	1.78 (1.43, 2.23)	88.1
Disease stage	14	5678	1.98 (1.60, 2.43)	85.7
Subgroup analyses				
Geographic region				
Asia	6	1511	2.26 (1.72, 2.97)	55.9
Europe/UK	3	1436	2.02 (1.11, 3.68)	80.6
US/Canada	5	1805	1.34 (1.12, 1.61)	34.1
NR/other	2	1473	1.67 (1.46, 1.92)	0
Follow‐up time				
<1 year	4	1347	2.32 (1.37, 3.92)	72.1
1–3 years	7	1700	2.15 (1.68, 2.75)	56.6
>3 years	3	1916	1.27 (0.95, 1.69)	68.4
NR	2	1262	1.29 (1.13, 1.46)	0
Cachexia definition				
IC criteria	11	3370	2.26 (1.80, 2.83)	73.0
WL ≥ 5%	5	2855	1.28 (1.12, 1.46)	39.7

Abbreviations: CI, confidence interval; HR, hazard ratio; I^2^, heterogeneity statistic; IC, international consensus; NR, not reported; REM, random‐effects model; UK, United Kingdom; US, United States; WL, weight loss.

#### Subgroup analyses

The validity of the base‐case meta‐analysis was further assessed by accounting for differences in study characteristics using subgroup analyses where studies were stratified according to geographic region, length of follow‐up time and definition of cachexia or weight loss used to define the study populations. All subgroup analyses showed a statistically significant association between cachexia or weight loss ≥ 5% and inferior survival (*Table* *4* and *Figures* *S3* and *S4*), with the exception of the subgroup analysis of studies with follow‐up time > 3 years, which was nonsignificant in the random‐effects model (HR [95% CI]: 1.27 [0.95, 1.69]; I^2^ = 68.4%) (*Table* *4* and *Figure* *S3* *B*) but reached significance in the fixed‐effects model (HR [95% CI]: 1.47 [1.30, 1.66]). However, the overall consistency of these subgroup analyses with the base‐case analysis indicated that the meta‐analysis results were robust and generalizable across variability in these parameters. Notably, the results of the subgroup analysis where studies were stratified according to use of the international consensus criteria for cachexia (HR [95% CI]: 2.26 [1.80, 2.83]) or weight loss ≥ 5% (HR [95% CI]: 1.28 [1.12, 1.46]) were consistent with the base‐case analysis (*Table* *4* and *Figure* *S4*).

#### Analyses of time‐varying effects, study influence and publication bias

Cumulative meta‐analyses conducted to identify time‐varying effects across studies consisted of separate analyses by data collection time period and publication year, with each study added in turn. As the data collection time periods overlapped across studies, the impact of this variable was assessed by calculating the midpoint of the data collection time period for each study for use in the cumulative meta‐analysis. Across studies, the midpoint of the data collection time period ranged from 2000 to 2019, and the publication year ranged from 2016 to 2021. These cumulative meta‐analyses did not identify any time‐varying effects across studies, indicating that the meta‐analysis results were robust to the different timeframes of data collection and publication.

A meta‐influence analysis was conducted to assess the relative influence of each study on the meta‐analysis results, in which each study was omitted in turn to assess the impact of excluding individual studies on the overall results. This analysis did not identify any potential outlier studies (lowest HR [95% CI]: 1.68 [1.40, 2.01]; highest HR [95% CI]: 1.90 [1.54, 2.34]), thus supporting the results of the base‐case meta‐analysis.

**Figure 4:**
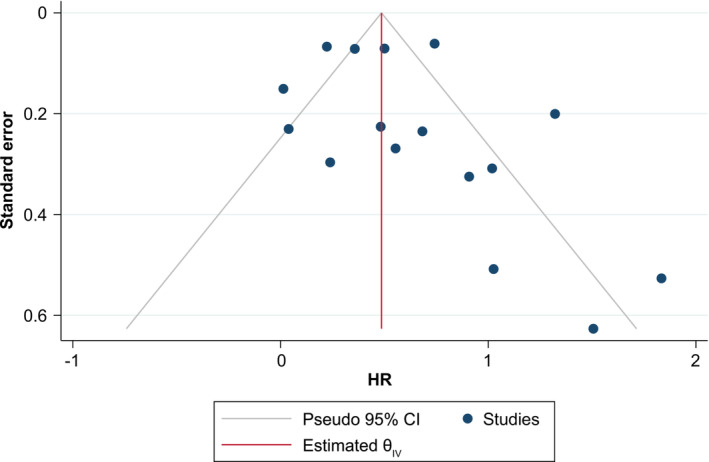
Funnel plot analysis for the base‐case meta‐analysis of the association between cachexia or weight loss ≥ 5% and overall survival in non‐small‐cell lung cancer. CI, confidence interval; HR, hazard ratio; θ, overall effect estimate.

A funnel plot analysis showed no indication of substantial publication bias across studies included in the meta‐analysis (*Figure* *4*). Moreover, as all the included studies had ≥ 100 patients, the potential for a small‐study effect was limited.

## Discussion

### Main findings

Cachexia, though highly prevalent^6^, ^7^, ^8^, ^9^ and a predictor of poorer prognosis^3^, ^4^ in NSCLC, is understudied and not routinely assessed. This SLR and the subsequent meta‐analysis were undertaken to provide an updated overview of the impact of cancer‐associated cachexia or weight loss on overall survival in adult patients with NSCLC. Sixteen studies (*n* = 6225 patients) conducted across a range of geographic locations, clinical settings and patient populations that assessed the association between cachexia—as defined by the international consensus criteria^1^—or any weight loss ≥ 5% and overall survival in the context of NSCLC were identified for inclusion in the base‐case meta‐analysis. This analysis demonstrated that cachexia, defined by these combined criteria, was associated with an 82% higher relative risk of mortality when compared with no cachexia or weight loss < 5% in patients with NSCLC (HR [95% CI]: 1.82 [1.47, 2.25]). Notably, when these criteria were analysed separately, the use of the international consensus criteria for cachexia defined a subgroup of patients at greater risk of mortality (HR [95% CI]: 2.26 [1.80, 2.83]). However, patients identified on the basis of weight loss ≥ 5%, but otherwise not meeting the international consensus criteria, were also found to have an increased risk of inferior overall survival (HR [95% CI]: 1.28 [1.12, 1.46]). Based on rigorous methodological, clinical and statistical heterogeneity assessments, including sensitivity and subgroup analyses, cumulative meta‐analyses and a meta‐influence analysis, the results of the meta‐analysis were considered to be robust.

### Context and key recommendations

To our knowledge, this is one of the few recent SLRs and meta‐analyses examining the impact of cachexia on overall survival in NSCLC to have focused the study selection process on those studies that used the international consensus criteria for cachexia^1^ to identify cachexia in their patient populations rather than individual features of cachexia, such as sarcopenia. Sarcopenia is a disorder of muscle weakness and muscle loss that can occur primarily due to aging or inactivity, or secondarily to pathologies like cancer. Though significant overlap exists between sarcopenia and cachexia, cachexia is a distinct entity due to inflammation and hypermetabolism that result in both fat and muscle loss and significant body weight loss. However, two recent analyses^18^, ^20^ assessing the impact of sarcopenia on cancer outcomes found sarcopenia to be associated with poor survival outcomes, including in patients with NSCLC. Buentzel et al.^18^ examined sarcopenia as a potential prognostic factor in 2521 patients with lung cancer across 15 studies that included patients with NSCLC (*n* = 13 studies), patients with SCLC (*n* = 1) and patients with NSCLC or SCLC (*n* = 1). In separate analyses of studies that used univariate and/or multivariate analysis, sarcopenia was associated with a two‐ to three‐fold higher risk of mortality (univariate analyses, HR [95% CI]: 1.96 [1.49, 2.59]; multivariate analyses, HR [95% CI]: 3.13 [2.06, 4.76]) in patients with lung cancer.^18^ Similarly, Takenaka et al.^20^ assessed the association between sarcopenia status and oncologic outcomes in 2501 patients with cancer treated with immune checkpoint inhibitors across 26 studies. In a subset of 551 patients (*n* = 6 studies) with NSCLC, sarcopenia was found to be significantly associated with poor overall survival (HR [95% CI]: 1.61 [1.19, 2.18]).^20^ Studies included in these meta‐analyses assessed sarcopenia primarily through the use of computed tomography.

Although our meta‐analysis set out to assess studies where the definition of cachexia was based on the international consensus criteria,^1^ which includes various anthropometric measures of skeletal muscle mass indicative of sarcopenia in addition to weight loss and reduced BMI, the majority of included studies (*n* = 12) used weight loss ≥ 5% as the sole criterion to identify patients with cachexia in their respective cohorts, with a few including additional components such as BMI (*n* = 4) and biochemical markers (*n* = 1). None of the studies included in this analysis included quantitative measures of muscle mass to identify patients with cachexia. Nevertheless, this analysis demonstrated that cachexia, primarily identified by the ≥ 5% weight loss cut point specified in international consensus criteria, was associated with an approximately two‐fold increase in the risk of mortality compared with no cachexia or weight loss < 5%. Thus, this simple measure of body composition defined a subgroup of patients with NSCLC at significantly greater risk of poorer overall survival. As such, we recommend that the measurement and recording of body weight in patient electronic records be routinely undertaken at all clinic visits so trends in body weight changes that might indicate or portend the onset of cachexia can be easily observed and acted upon at the earliest opportunity. Furthermore, weight loss should be routinely considered in the context of clinical trials of anti‐cachexia interventions, both in terms of the eligibility criteria for trial entry and as a trial endpoint. This will help facilitate the meaningful translation of clinical trial results to the clinic without placing an undue burden on patients participating in these trials.^52^

The importance of monitoring weight loss as a prognostic indicator of inferior survival in NSCLC is underscored by the recent publication of two large, retrospective studies that assessed the impact of weight loss ≥ 5% on survival outcomes in patients with advanced lung cancer^53^, ^54^ published since our SLR was conducted. First, an analysis of 10 128 patients with advanced NSCLC (*n* = 7321) or SCLC (*n* = 2807) from 63 National Cancer Institute (NCI)‐sponsored trials in the United States found that weight loss > 5% over the previous 3 or 6 months (depending on the trial) was associated with decreased overall survival compared with weight loss ≤ 5% (HR [95% CI]: 1.20 [1.14, 1.26]).^53^ Notably, per cent weight loss had a more substantial impact on survival than BMI, particularly in the subgroup of patients with NSCLC.^53^ Second, an analysis of a large Japanese database of patients with advanced lung cancer (*n* = 12 320; *n* = 8489 with weight loss data) found that weight loss ≥ 5% over the previous 6 months was, again, associated with reduced overall survival (HR [95% CI]: 1.37 [1.27, 1.47]).^54^ Moreover, although the use of immunotherapy has led to marked improvements in the prognosis and survival of patients with advanced NSCLC,^55^ recent analyses have demonstrated an association between weight loss ≥ 5% and treatment failure with immune checkpoint inhibitors.^39^, ^41^, ^44^, ^56^, ^57^ Again, this emphasizes the importance of routinely assessing body weight and reviewing any observed changes in body weight during treatment of patients with NSCLC.

### Strengths and limitations

This meta‐analysis has a number of strengths. First, limiting the timeframe of the SLR to include only those studies published within the 5 years prior to the date the literature searches were conducted increases the likelihood that the findings of the meta‐analysis reflect the increased effectiveness of current treatment regimens for NSCLC (including immunotherapy and targeted therapies) and increased use of the international consensus criteria to define cachexia. It should be noted, however, that although the publication date ranged from 2016 to 2021, the data collection time period of the included studies spanned a timeframe of > 30 years (1987–2020). Second, included studies were restricted to those enrolling ≥ 100 patients in total and using multivariate analyses, thus minimizing the potential for small‐study effects and confounding. Third, the sample size available for inclusion in the meta‐analysis was large—6225 patients with NSCLC and overall survival data across 16 studies—meaning the data were representative of a range of clinical settings and patient populations. Fourth, although unintended, the use of the same diagnostic measure (weight loss) and cut point (≥ 5%) to define cachexia across studies likely had a benefit in reducing the statistical heterogeneity often associated with conducting a meta‐analysis of studies that use different methods of measurement and cut point thresholds.

This meta‐analysis also has some limitations. First, all 16 studies included in the meta‐analysis were observational in nature, and, as such, the analysis may be subject to the same confounding factors as the original studies. Also, the majority of the included studies (*n* = 14) collected patient data retrospectively. The use of archived patient records can result in a high proportion of missing data on individual component criteria for cachexia (e.g., BMI and muscle mass assessment) and survival outcomes. As noted above, this may be reflected in the criteria for cachexia used by the studies included in this meta‐analysis, with the majority of studies using weight loss only to identify the population of interest. Second, this meta‐analysis was based on study‐level data rather than individual patient‐level data. As such, more detailed subgroup analyses and investigations of the effect of cachexia progression during follow‐up were not possible. Third, a high level of statistical heterogeneity was identified across the studies included in the base‐case analysis, which likely was due to observed differences in the characteristics of the study populations (age, gender, performance status and disease stage) and of the studies themselves (geographic region, data collection time period, follow‐up time and treatment type). However, given the directional alignment of the HRs of the individual studies and the pooled estimate, the considerable overlap in the CIs across individual studies and the concordance of sensitivity and subgroup analyses with the primary meta‐analysis outcome, the impact of this heterogeneity is likely to be limited. Indeed, the consistency of the results obtained across the various assessments of methodological, clinical and statistical heterogeneity suggests that the results of the analysis are robust and generalizable across a range of clinical settings and patient populations. Fourth, the SLR was limited to searches conducted in Embase and PubMed to identify articles published in English, so studies published in journals not indexed in these databases or in another language, or published after the date the searches were run (11 October 2021), would have been missed.

## Conclusions

This meta‐analysis has demonstrated that cachexia, as defined by the international consensus diagnostic criteria,^1^ or weight loss ≥ 5% were associated with a significantly higher risk of mortality in patients with NSCLC when compared with no cachexia or weight loss < 5%. Based on rigorous heterogeneity assessments, the results of the meta‐analysis were considered to be robust. This analysis highlights the importance of early awareness of cachexia in NSCLC, which could facilitate the timely implementation of symptom management strategies and improve patient outcomes. Also, it would enhance the identification of patients who are candidates for participation in cachexia clinical trials. This could be achieved by more standardized reporting of cachexia parameters in routine clinical practice and ongoing clinical trials. At a minimum, performing weight measurements and entering results in electronic medical records at every clinic visit will provide benefits in the treatment of individual patients and for ongoing clinical trials evaluating new treatment strategies for cachexia.

## Conflict of interest statement

Philip D. Bonomi reports honoraria from Pfizer, Helsinn Healthcare and Roche Genentech. Jeffrey Crawford reports consulting/advisory roles for and honoraria from Actimed Therapeutics, AVEO, Enzychem Lifesciences, Faraday Pharmaceuticals, G1 Therapeutics, Merck, Partner Therapeutics, Pfizer, Sandoz, BIO Alta and Seagen and research funding from Helsinn Healthcare, AstraZeneca and Pfizer (paid to his institution). Richard F. Dunne reports honoraria from Helsinn Healthcare, Exelixis, Toray Industries and Merck. Eric J. Roeland reports participation in scientific advisory boards for Napo Pharmaceuticals, Care4ward, Actimed Therapeutics, Meter Health, Alerion and Takeda; consulting roles for Veloxis Therapeutics and BYOMass; and participation in data safety monitoring boards for Enzychem Lifesciences. Karen E. Smoyer is an employee of Curo, part of the Envision Pharma Group, who were paid consultants to Pfizer in relation to this work. Mohd Kashif Siddiqui is an employee of EBM Health Consultants, who were paid consultants to Envision Pharma Group in relation to this work. Thomas D. McRae and James H. Revkin were employees of Pfizer at the time of the analysis and may hold stock or stock options in Pfizer. Michelle I. Rossulek and Lisa C. Tarasenko are employees of Pfizer and may hold stock or stock options in Pfizer.

## Supporting information


**Table S1.** Embase search strategy.
**Table S2.** PubMed search strategy.
**Table S3.** Analyses performed on NSCLC studies identified for inclusion in the meta‐analysis, including sensitivity and subgroup analyses.
**Table S4.** Quality assessment using the Newcastle–Ottawa Scale^a^ of NSCLC studies identified for inclusion in the meta‐analysis (*n* = 16).
**Figure S1.** PRISMA diagram of the literature‐screening process for the broader SLR on cachexia in selected solid‐tumor cancers.
**Figure S2.** Sensitivity meta‐analyses of the association between cachexia or weight loss ≥ 5% and overall survival in NSCLC based on (a) age, (b) gender, (c) performance status, and (d) disease stage: Random‐effects models.
**Figure S3.** Subgroup meta‐analyses of the association between cachexia or weight loss ≥ 5% and overall survival in NSCLC stratified by (a) geographic region and (b) length of follow‐up time: Random‐effects models.
**Figure S4.** Subgroup meta‐analysis of the association between cachexia or weight loss ≥ 5% and overall survival in NSCLC stratified by cachexia definition: Random‐effects model.
