# Supplementary material for: Mortality burden of pre‐treatment weight loss in patients with non‐small‐cell lung cancer: A systematic literature review and meta‐analysis
Source: J Cachexia Sarcopenia Muscle. 2024 Apr 22;15(4):1226–39. doi: 10.1002/jcsm.13477 (PMC11294038; doi:10.1002/jcsm.13477)
Supplement: Supplementary file 1 — Table S1. Embase search strategy. Table S2. PubMed search strategy. Table S3. Analyses performed on NSCLC studies identified for inclusion in the meta‐analysis, including sensitivity and subgroup analyses. Table S4. Quality assessment using the Newcastle–Ottawa Scalea of NSCLC studies identified for inclusion in the meta‐analysis (n = 16). Figure S1. PRISMA diagram of the literature‐screening process for the broader SLR on cachexia in selected solid‐tumor cancers. Figure S2. Sensitivity meta‐analyses of the association between cachexia or weight loss ≥ 5% and overall survival in NSCLC based on (a) age, (b) gender, (c) performance status, and (d) disease stage: Random‐effects models. Figure S3. Subgroup meta‐analyses of the association between cachexia or weight loss ≥ 5% and overall survival in NSCLC stratified by (a) geographic region and (b) length of follow‐up time: Random‐effects models. Figure S4. Subgroup meta‐analysis of the association between cachexia or weight loss ≥ 5% and overall survival in NSCLC stratified by cachexia definition: Random‐effects model. [file JCSM-15-1226-s001.docx]

**SUPPORTING INFORMATION**

**Mortality burden of pre-treatment weight loss in patients with non-small-cell lung cancer: A systematic literature review and meta-analysis**

**Philip D. Bonomi^1^, Jeffrey Crawford^2^, Richard F. Dunne^3^, Eric J. Roeland^4^, Karen E. Smoyer^5^, Mohd Kashif Siddiqui^6^, Thomas D. McRae^7^*, Michelle I. Rossulek^8^, James H. Revkin^9^*, and Lisa C. Tarasenko^10^**

*^1^Department of Internal Medicine, Division of Hematology, Oncology and Cell Therapy, Rush University Medical Center, Chicago, IL, USA; ^2^Duke Cancer Institute, Duke University Medical Center, Durham, NC, USA; ^3^Department of Medicine and Wilmot Cancer Institute, Division of Hematology/Oncology, University of Rochester Medical Center, Rochester, NY, USA; ^4^Knight Cancer Institute, Oregon Health and Science University, Portland, OR, USA; ^5^Curo^,^ Envision Pharma Group, Philadelphia, PA, USA; ^6^EBM Health Consultants, New Delhi, Delhi, India; ^7^Internal Medicine Business Unit, Global Product Development, Pfizer Inc, New York, NY, USA; ^8^Internal Medicine Research Unit, Worldwide Research, Development and Medical, Pfizer Inc, Cambridge, MA, USA; ^9^Internal Medicine Research Unit, Clinical Development, Pfizer Inc, Cambridge, MA, USA; ^10^Global Medical Affairs, Pfizer Inc, New York, NY, USA*

**Affiliation at the time the analysis was conducted*

**Correspondence:** Philip D. Bonomi MD, MS, Department of Internal Medicine, Division of Hematology, Oncology and Cell Therapy, Rush University Medical Center, 1725 W. Harrison St, Suite 809, Chicago 60612, IL, USA

**Table S1.** Embase search strategy.

| **Step** | **Search string** |
| --- | --- |
| 1 | exp cachexia/ or exp sarcopenia/ |
| 2 | (cachexia or sarcop?nia).ti,ab. |
| 3 | (emaciation or wasting or weight loss or weight reduction).ti,ab |
| 4 | 1 or 2 or 3 |
| 5 | (neoplasm$ or glioblastoma$ or cancer* or tumour$ or tumor$ or malignan$ or carcinoma$ or adenocarcinoma$ or oncolog$ or gliom$).ti,ab |
| 6 | 4 and 5 |
| 7 | (surviv* or mortality or death*).ti,ab,kw |
| 8 | 6 and 7 |
| 9 | (animal$ not human$).sh,hw. |
| 10 | conference abstract.pt |
| 11 | (editorial or comment* or letter or note or case study or case studies or case report).pt. or (editorial/ or letter/ or case study/ or case report/ or note/) or (case report* or case stud*).ti,ab |
| 12 | (infan* or child* or adolescen* or pediatr* or paediatr* or juvenile).ti,ab,kw. |
| 13 | or/9–12 |
| 14 | 8 not 13 |
| 15 | limit 14 to English language |
| 16 | limit 16 to yr="2011–Current" |

**Table S2.** PubMed search strategy.

| **Step** | **Search string** |
| --- | --- |
| 1 | "cachexia"[MeSH Terms] OR "cachexia"[All Fields] OR "cachexias"[All Fields] OR "sarcopenia"[MeSH Terms] OR "emaciation"[Title/Abstract] OR "wasting"[Title/Abstract] OR "weight loss"[Title/Abstract] OR "weight reduction"[Title/Abstract] OR "sarcopenia"[Title/Abstract] OR "sarcopaenia"[Title/Abstract] |
| 2 | "neoplasms"[MeSH Terms] OR "neoplasms"[All Fields] OR "neoplasm"[All Fields] OR "glioblastoma"[All Fields] OR "glioblastomas"[All Fields] OR "cancerous"[All Fields] OR "cancer"[All Fields] OR "cancers"[All Fields] OR "tumour"[All Fields] OR "tumor"[All Fields] OR "tumours"[All Fields] OR "tumors"[All Fields] OR "malign"[All Fields] OR "malignant"[All Fields] OR "malignancies"[All Fields] OR "malignancy"[All Fields] OR "carcinoma"[MeSH Terms] OR "carcinoma"[All Fields] OR "carcinomas"[All Fields] OR "carcinoma s"[All Fields] OR "adenocarcinoma"[MeSH Terms] OR "adenocarcinoma"[All Fields] OR "adenocarcinomas"[All Fields] OR "oncology"[All Fields] OR "glioma"[MeSH Terms] OR "glioma"[All Fields] OR "gliomas"[All Fields] |
| 3 | 1 and 2 |
| 4 | "survival"[Title/Abstract] OR "mortality"[Title/Abstract] OR "death"[Title/Abstract] |
| 5 | 3 and 4 |
| 6 | "editorial"[Publication Type] OR "comment"[Publication Type] OR "letter"[Publication Type] OR "case reports"[Publication Type] OR "animal*"[Title/Abstract] OR "in vitro"[Title/Abstract] OR "tissue*"[Title/Abstract] OR "murine"[Title/Abstract] OR "mouse"[Title/Abstract] OR "mice"[Title/Abstract] OR "swine*"[Title/Abstract] OR "pig"[Title/Abstract] OR "pigs"[Title/Abstract] OR "porcine"[Title/Abstract] OR "rat"[Title/Abstract] OR "rats"[Title/Abstract] OR "rodent*"[Title/Abstract] OR "monkey"[Title/Abstract] OR "monkeys"[Title/Abstract] OR "ape"[Title/Abstract] OR "apes"[Title/Abstract] OR "dog"[Title/Abstract] OR "dogs"[Title/Abstract] OR "canine*"[Title/Abstract] OR "cat"[Title/Abstract] OR "cats"[Title/Abstract] OR "feline*"[Title/Abstract] OR "cow"[Title/Abstract] OR "bovine"[Title/Abstract] OR "horse"[Title/Abstract] OR "equine"[Title/Abstract] OR "infant"[Title/Abstract] OR "infants"[Title/Abstract] OR "child"[Title/Abstract] OR "children"[Title/Abstract] OR "adolescent"[Title/Abstract] OR "adolescents"[Title/Abstract] OR "adolescence"[Title/Abstract] OR "pediatric"[Title/Abstract] OR "paediatric"[Title/Abstract] OR "juvenile"[Title/Abstract] |
| 7 | 5 not 6 |
| 8 | Limit to English language |
| 9 | Limit from 2011–2021 |

**Table S3.** Analyses performed on NSCLC studies identified for inclusion in the meta-analysis, including sensitivity and subgroup analyses.

| **Type of Analysis/Variable** | **Inclusion, Exclusion, and Stratification of Studies for Analysis** |
| --- | --- |
| Base-case analysis | Included: All 16 studies irrespective of differences in study and baseline characteristics |
| Sensitivity analyses | |
| Age | Excluded: Topkan *et al.* 2020 [49] (pts aged ≤45 yrs) |
| Gender | Excluded: Ganti *et al.* 2019 [33], Patel *et al.* 2017 [43] (lower proportion of males); Moumtzi *et al.* 2016 [42] (higher proportion of males) |
| Performance status | Excluded: Jouinot *et al.* 2020 [36], Lee *et al.* 2020 [38], Moore *et al.* 2020 [40], Roch *et al.* 2020 [44] (higher proportion of pts with PS ≥ 2) |
| Disease stage | Excluded: Holmes *et al.* 2017 [34], Moore *et al.* 2020 [40] (stage I/II pts only) |
| Subgroup analyses | |
| Geographic region | Stratified: North America, Europe, Asia, NR/other |
| Follow-up time | Stratified: <1 yr, 1–3 yrs, >3 yrs, NR |
| Cachexia definition | Stratified: IC criteria for cachexia, WL ≥ 5% but not other IC criteria |
| Cumulative analyses | |
| Data collection time period | Variable: Mid-point of the data collection time period; each study added in turn |
| Publication year | Variable: Year of publication; each study added in turn |
| Meta-influence analysis | Each study omitted in turn |

IC, international consensus; NR, not reported; PS, performance status; pts, patients; WL, weight loss; yr(s), year(s).

**Table S4.** Quality assessment using the Newcastle–Ottawa Scale^a^ of NSCLC studies identified for inclusion in the meta-analysis (n = 16).

| **Author; Year** | **Selection** | | | | **Comparability** | **Outcome** | | | **Total** |
| --- | --- | --- | --- | --- | --- | --- | --- | --- | --- |
|  | **Representative-ness of the exposed cohort** | **Selection of the non-exposed cohort** | **Ascertainment of exposure** | **Demonstration that outcome of interest was not present at start of study** | **Comparability of cohorts on the basis of the design or analysis** | **Assessment of outcome** | **Was follow up long enough for outcomes to occur?** | **Adequacy of follow up of cohorts** | **Score** |
| Gannavarapu et al.; 2018 [32] | 0 | 1 | 1 | 1 | 2 | 1 | 1 | 1 | 8 |
| Ganti et al.; 2019 [33] | 1 | 1 | 1 | 1 | 2 | 1 | 1 | 1 | 9 |
| Holmes et al.; 2017 [34] | 0 | 1 | 1 | 1 | 2 | 1 | 1 | 1 | 8 |
| Jo et al.; 2021^b^ [35] | 0 | 1 | 1 | 1 | 2 | 1 | 1 | 1 | 8 |
| Jouinot et al.; 2020 [36] | 0 | 0 | 1 | 1 | 2 | 1 | 1 | 1 | 7 |
| Lee et al.; 2020 [38] | 0 | 1 | 1 | 1 | 2 | 1 | 1 | 1 | 8 |
| Miyawaki et al.; 2020 [39] | 0 | 1 | 1 | 1 | 2 | 1 | 1 | 1 | 8 |
| Moore et al.; 2020 [40] | 1 | 0 | 1 | 1 | 2 | 1 | 1 | 1 | 8 |
| Morimoto et al.; 2021 [41] | 1 | 1 | 1 | 1 | 2 | 1 | 1 | 1 | 9 |
| Moumtzi et al.; 2016 [42] | 0 | 1 | 1 | 1 | 2 | 1 | 1 | 1 | 8 |
| Patel et al.; 2017 [43] | 0 | 1 | 1 | 1 | 2 | 1 | 1 | 1 | 8 |
| Roch et al.; 2020 [44] | 0 | 1 | 1 | 1 | 2 | 1 | 1 | 1 | 8 |
| Topkan et al.; 2018 [48] | 0 | 0 | 1 | 1 | 2 | 1 | 1 | 1 | 7 |
| Topkan et al.; 2020 [49] | 0 | 1 | 1 | 1 | 2 | 1 | 1 | 1 | 8 |
| Wang et al.; 2017 [50] | 0 | 1 | 1 | 1 | 2 | 1 | 1 | 1 | 8 |
| Watte et al.; 2018 [51] | 0 | 1 | 1 | 1 | 2 | 1 | 1 | 1 | 8 |

^a^ Information on the Newcastle–Ottawa Scale is available in Ref. [31]. Score: ≥7, good; 4–6, fair; 0–3, poor.

^b^ E-publication date: June 2021; final publication date: February 2022.

**Figure S1.** PRISMA diagram of the literature-screening process for the broader SLR on cachexia in selected solid-tumor cancers.


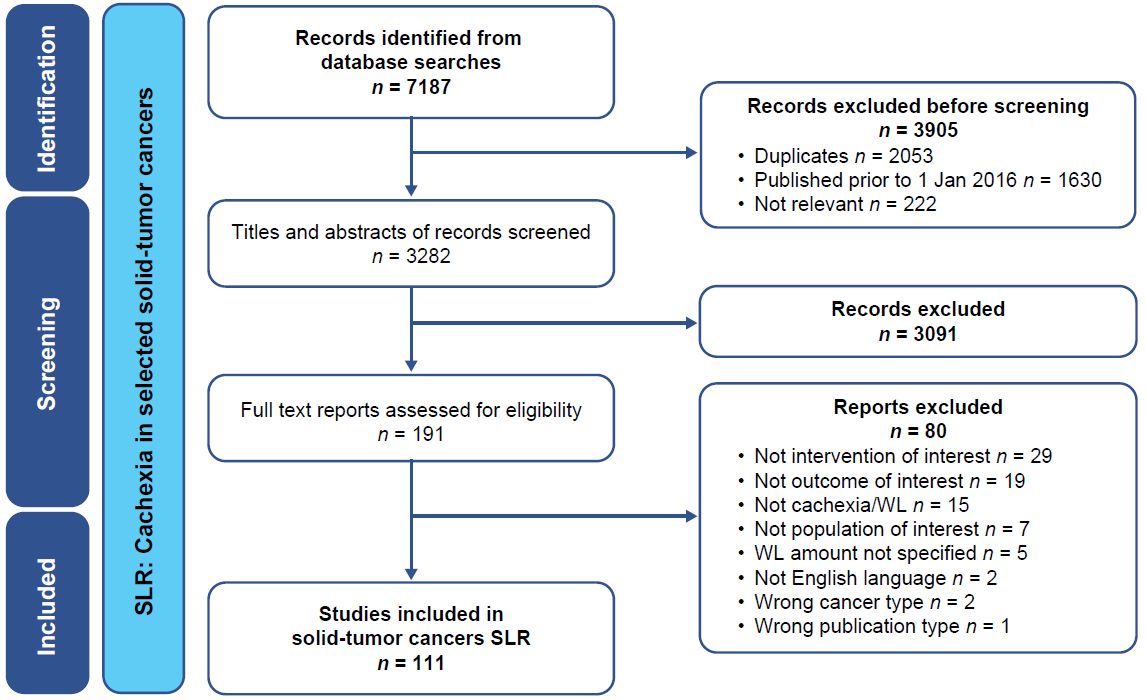


PRISMA, Preferred Reporting Items for Systematic Reviews and Meta-Analyses; SLR, systematic literature review; WL, weight loss.

**Figure S2.** Sensitivity meta-analyses of the association between cachexia or weight loss ≥ 5% and overall survival in NSCLC based on (a) age, (b) gender, (c) performance status, and (d) disease stage: Random-effects models.


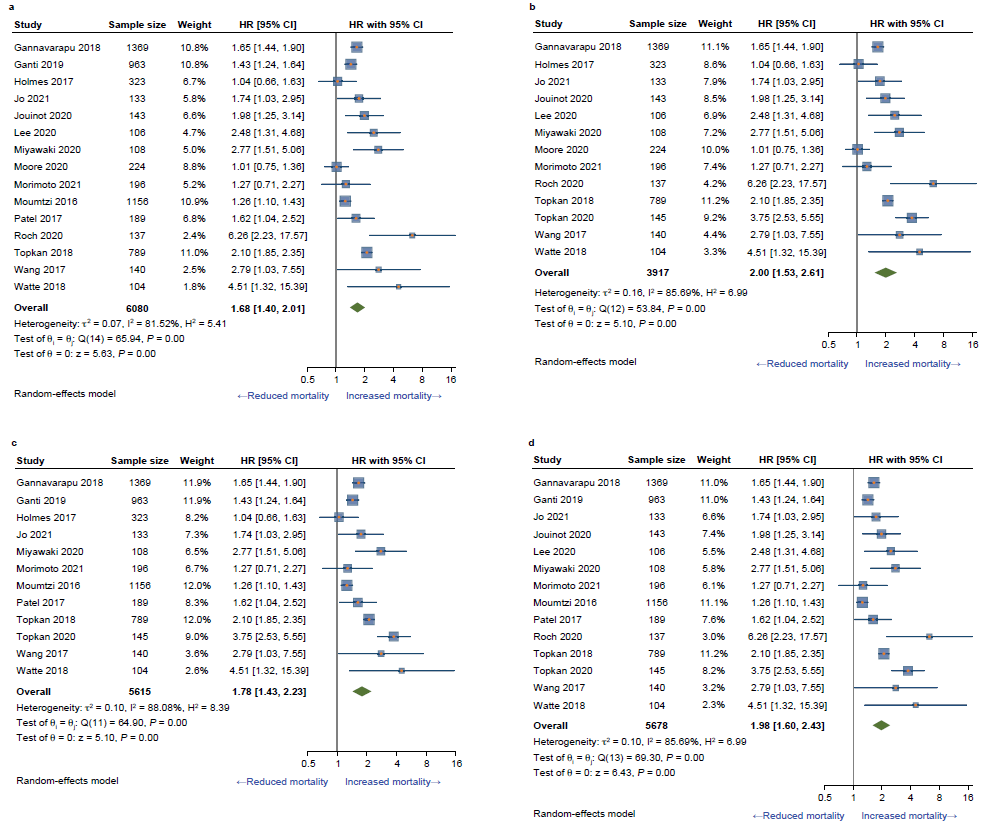


Sample size reflects patients with NSCLC and OS data, which for some studies is less than the number of patients with baseline data.

CI, confidence interval; H^2^, homogeneity statistic; HR, hazard ratio; I^2^, heterogeneity statistic; OS, overall survival; Q, Cochrane Q (Chi-square statistic); T^2^, tau-square; θ, overall effect estimate; z, normality distribution.

**Figure S3.** Subgroup meta-analyses of the association between cachexia or weight loss ≥ 5% and overall survival in NSCLC stratified by (a) geographic region and (b) length of follow-up time: Random-effects models.


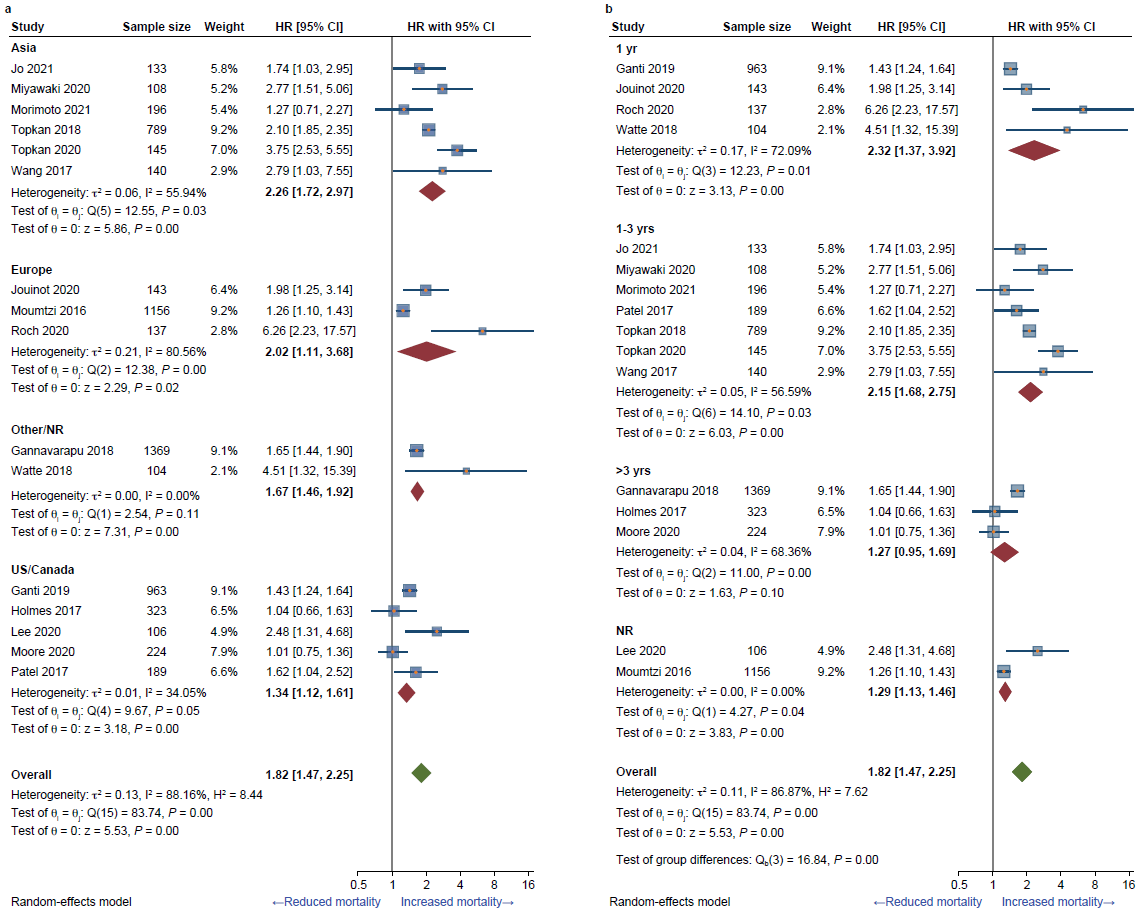


Sample size reflects patients with NSCLC and OS data, which for some studies is less than the number of patients with baseline data.

CI, confidence interval; H^2^, homogeneity statistic; HR, hazard ratio; I^2^, heterogeneity statistic; NR, not reported; OS, overall survival; Q, Cochrane Q (Chi-square statistic); T^2^, tau-square; θ, overall effect estimate; US, United States; z, normality distribution.

**Figure S4.** Subgroup meta-analysis of the association between cachexia or weight loss ≥ 5% and overall survival in NSCLC stratified by cachexia definition: Random-effects model.


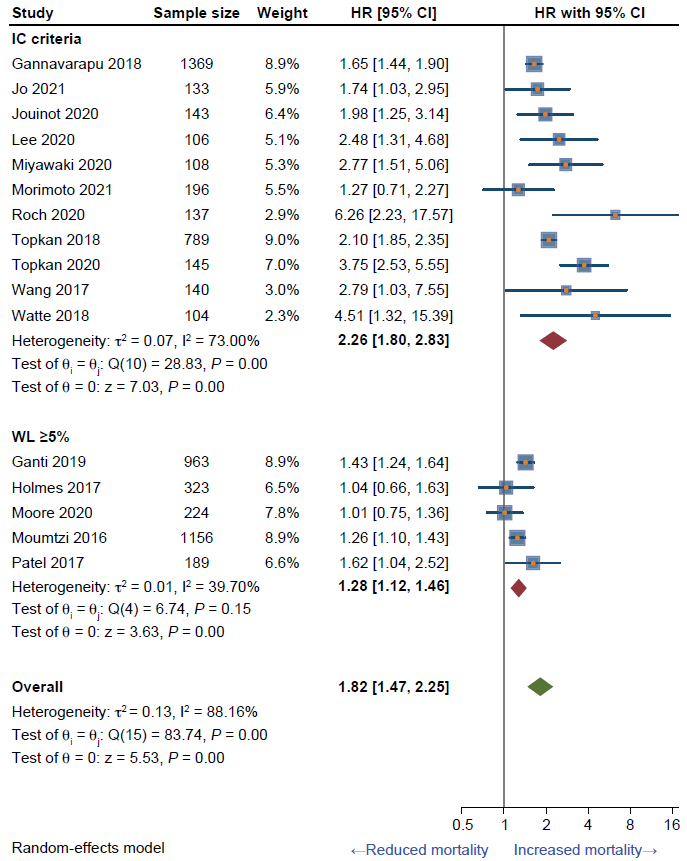


Sample size reflects patients with NSCLC and OS data, which for some studies is less than the number of patients with baseline data.

CI, confidence interval; H^2^, homogeneity statistic; HR, hazard ratio; I^2^, heterogeneity statistic; IC, international consensus; OS, overall survival; Q, Cochrane Q (Chi-square statistic); T^2^, tau-square; θ, overall effect estimate; WL, weight loss; z, normality distribution.
